# Supplementary material for: Seminoma and Embryonal Carcinoma Footprints Identified by Analysis of Integrated Genome-Wide Epigenetic and Expression Profiles of Germ Cell Cancer Cell Lines
Source: PLoS One. 2014 Jun 2;9(6):e98330. doi: 10.1371/journal.pone.0098330 (PMC4041891; doi:10.1371/journal.pone.0098330)
Supplement: File S1 — ZIP file containing DMRforPairs output for significant regions. Please start from the html files. (ZIP) [file pone.0098330.s008.zip › figures/24151.pdf]

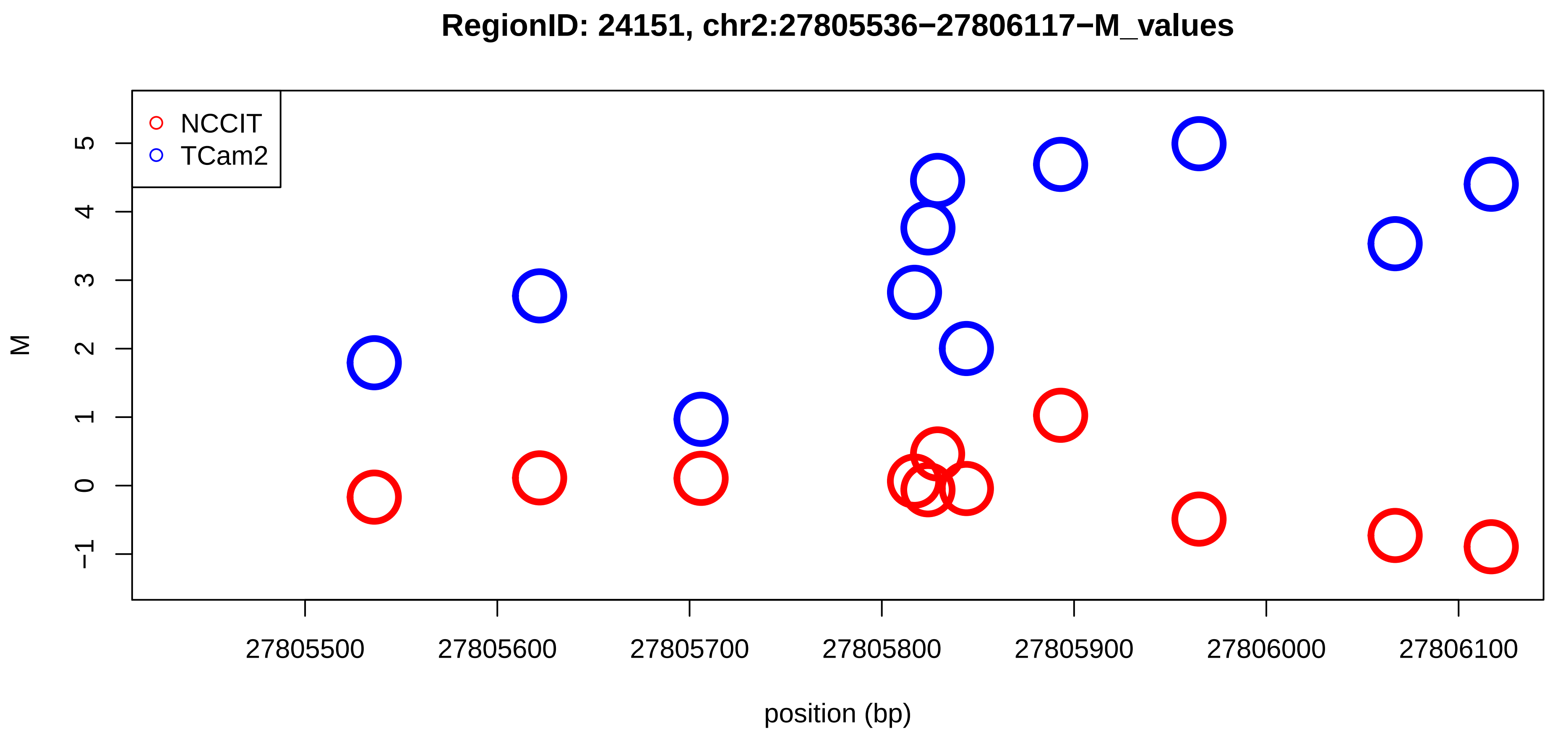
RegionID: 24151, chr2:27805536−27806117−M_values
NCCIT
TCam2
M
5
4
3
2
1
0
−1
27805500
27805600
27805700
27805800
27805900
27806000
27806100
position (bp)

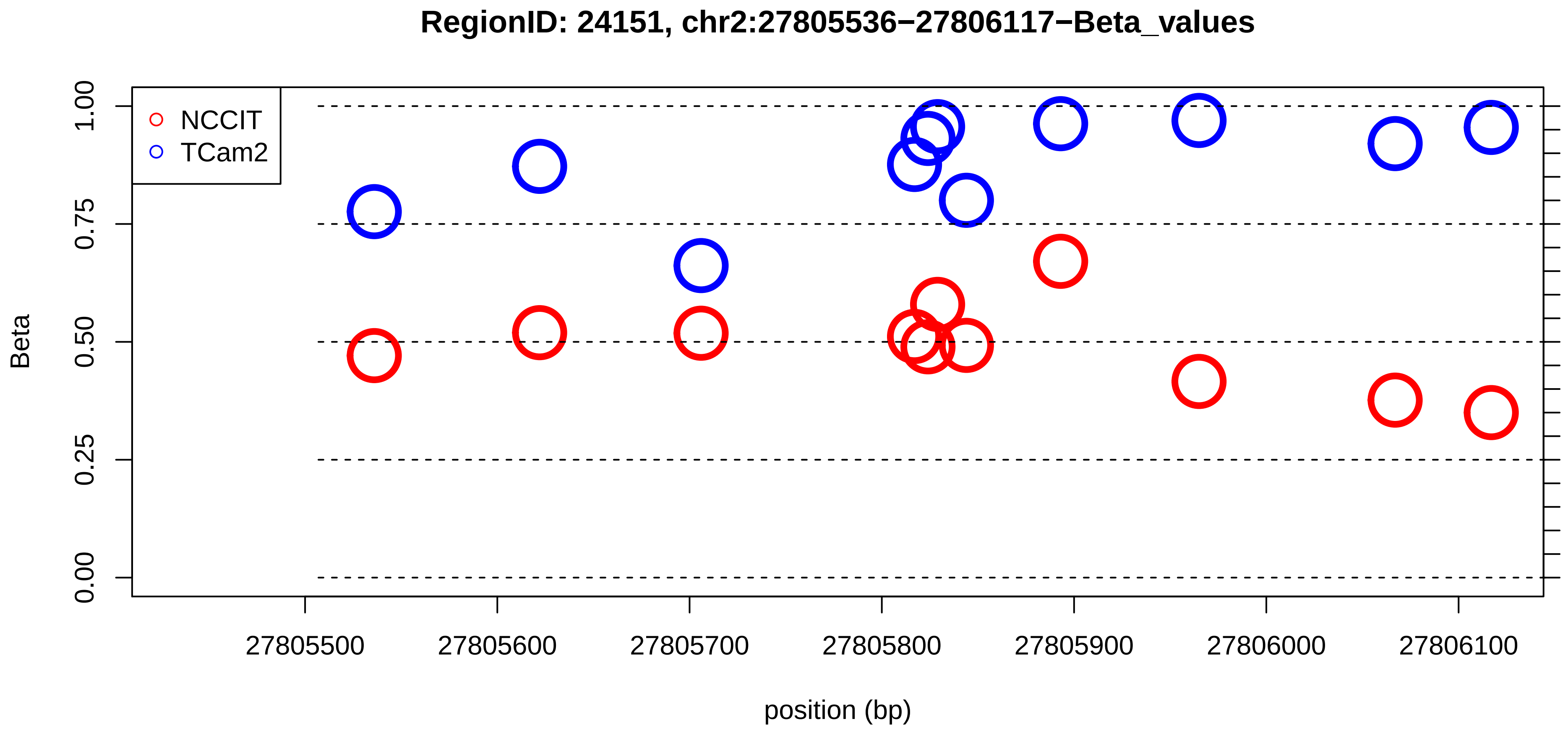
RegionID: 24151, chr2:27805536−27806117−Beta_values
NCCIT
TCam2
Beta
1.00
0.75
0.50
0.25
0.00
27805500
27805600
27805700
27805800
27805900
27806000
27806100
position (bp)

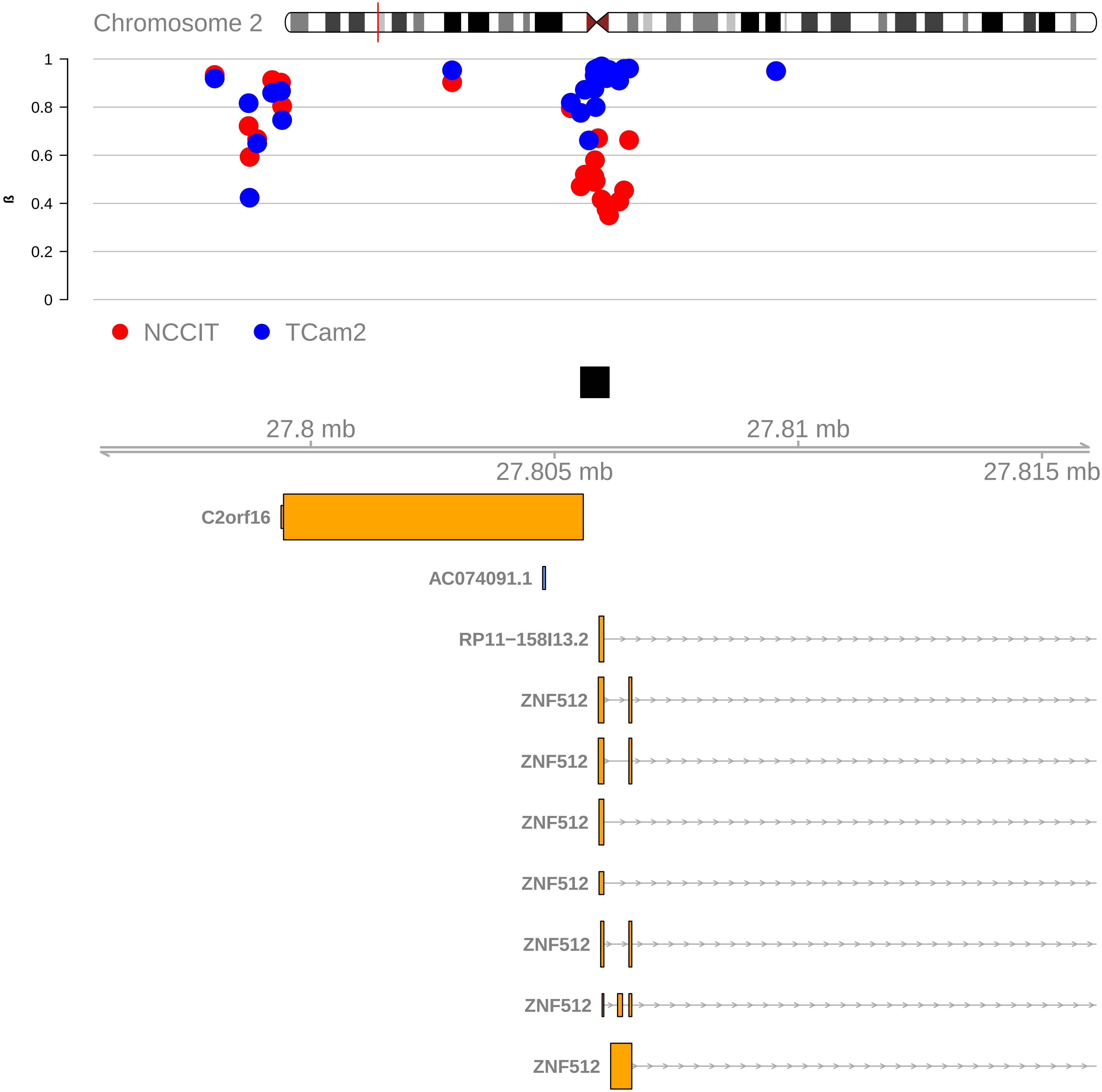

Chromosome 2
β
1
0.8
0.6
0.4
0.2
0
NCCIT
TCam2
27.8 mb
27.81 mb
27.805 mb
27.815 mb
C2orf16
AC074091.1
RP11−158I13.2
ZNF512
ZNF512
ZNF512
ZNF512
ZNF512
ZNF512
ZNF512
